# Immobilization of Gelatin on Fibers for Tissue Engineering Applications: A Comparative Study of Three Aliphatic Polyesters

**DOI:** 10.3390/polym14194154

**Published:** 2022-10-04

**Authors:** Oliwia Jeznach, Dorota Kołbuk, Tobias Reich, Paweł Sajkiewicz

**Affiliations:** 1 Institute of Fundamental Technological Research, Polish Academy of Sciences, Pawinskiego 5B, 02-106 Warsaw, Poland; 2 Department of Chemistry, Johannes Gutenberg University Mainz, Fritz-Strassmann-Weg 2, 55128 Mainz, Germany

**Keywords:** gelatin, aminolysis, surface modification, electrospinning

## Abstract

Immobilization of cell adhesive proteins on the scaffold surface has become a widely reported method that can improve the interaction between scaffold and cells. In this study, three nanofibrous scaffolds obtained by electrospinning of poly(caprolactone) (PCL), poly(L-lactide-co-caprolactone) (PLCL) 70:30, or poly(L-lactide) (PLLA) were subjected to chemical immobilization of gelatin based on aminolysis and glutaraldehyde cross-linking, as well as physisorption of gelatin. Two sets of aminolysis conditions were applied to evaluate the impact of amine group content. Based on the results of the colorimetric bicinchoninic acid (BCA) assay, it was shown that the concentration of gelatin on the surface is higher for the chemical modification and increases with the concentration of free NH2 groups. XPS (X-ray photoelectron spectroscopy) analysis confirmed this outcome. On the basis of XPS results, the thickness of the gelatin layer was estimated to be less than 10 nm. Initially, hydrophobic scaffolds are completely wettable after coating with gelatin, and the time of waterdrop absorption was correlated with the surface concentration of gelatin. In the case of all physically and mildly chemically modified samples, the decrease in stress and strain at break was relatively low, contrary to strongly aminolyzed PLCL and PLLA samples. Incubation testing performed on the PCL samples showed that a chemically immobilized gelatin layer is more stable than a physisorbed one; however, even after 90 days, more than 60% of the initial gelatin concentration was still present on the surface of physically modified samples. Mouse fibroblast L929 cell culture on modified samples indicates a positive effect of both physical and chemical modification on cell morphology. In the case of PCL and PLCL, the best morphology, characterized by stretched filopodia, was observed after stronger chemical modification, while for PLLA, there was no significant difference between modified samples. Results of metabolic activity indicate the better effect of chemical immobilization than of physisorption of gelatin.

## 1. Introduction

Understanding and improving cell–material interaction is a topic of fundamental importance in tissue engineering research [1]. At the early stage of the scaffold contact with culture medium or physiological environment, surface properties play the most significant role. Firstly, non-specific protein adsorption on the material surface occurs. This protein layer influences cell attachment, by providing or not, adhering sites for integrin-driven interaction [2,3]. Type and quantity of adsorbed proteins, and thus cell adhesion, depend on many material surface characteristics, such as charge, wettability, and electrostatic interaction [3,4,5,6]. Taking into account that cells adhere on the surface mainly via integrin–ligand binding, research effort is put into supplying the scaffolds with extracellular matrix (ECM)-originated adhesive proteins to enable specific cell attachment [7].

ECM molecules, such as fibronectin, vitronectin, or collagen, contain various peptide sequences that can be recognized by cell receptors. One of the most studied is the Arg-Gly-Asp (RGD) sequence, which can be provided by the denatured form of collagen–gelatin [8,9]. Depending on the type of protein, the RGD motif can be bound to different cell integrins, probably because of various spatial conformations of RGD associated with the vicinity of different amino acids or the existence of different synergistic sequences on ECM molecules [10]. In the case of gelatin, the RGD sequence is recognized by αvβ3 and α5β1 integrins [11]. Both integrins are present in, e.g., L929 cells [12,13]. Active integrins bound to adhesion molecules are attached to actin filaments through the focal adhesion complexes, which enable the transduction of mechanical signals from ECM to the cell [14,15]. Although specific integrin-mediated cell adhesion is widely reported in the literature of tissue engineering, there is still a need for studying factors, such as spatial conformation of ECM molecule [16,17] or so-called early cell adhesion preceding attachment of integrin to peptide sequence [18].

Currently, a lot of effort is put into the development of surface modification techniques to improve cell response to the scaffold without losing its mechanical properties or changing the degradability profile, as is observed in the case of bulk modification [19]. Electrospun nano- and submicron fibers made of aliphatic polyesters are widely studied in various tissue engineering applications (e.g., brain [20], tendon tissue regeneration [21], blood vessels [22], wound healing [23]), because of their biomimetic architecture, biocompatibility, atoxic degradation products, tunable mechanical properties, and reproducibility. However, from the perspective of the initial cell–scaffold contact, there is a problem with their hydrophobicity and lack of bioactive moieties, which could interact with cells [24]. Immobilization of biomolecules onto polyester fibers is achieved through various methods originating from chemical binding (e.g., methods based on aminolysis [25], hydrolysis [26], “click chemistry” [27]) or physical interaction between biomolecules and polymer (e.g., methods exploiting plasma treatment [28], layer-by-layer deposition [29], and simple surface entrapment [30]). In a review on recent advances in the modification of electrospun fibers, the authors pointed out many examples with a comprehensive presentation of modification method and obtained biological results [19]. Nowadays, there is a wide range of available methods, which improve cellular response, but it is important to consider their pros and cons, such as impact on bulk properties, complexity of process, etc. There is also too little information on long-term performance of the modified scaffolds under in vivo conditions. In our opinion, too little attention is also paid to fundamental understanding of mechanisms of the modification processes.

In this study, the immobilization of gelatin was investigated onto the surface of electrospun fibers made of three types of aliphatic polyesters: poly(caprolactone), poly(L-lactide-co-caprolactone), and poly(L-lactide). Aminolysis was applied in our research to introduce free amine functional groups on fibers’ surface as the “coupling sites” followed by glutaraldehyde cross-linking to chemically bind gelatin to the polymer. The polyesters used in our study differ in physicochemical properties, such as glass-transition temperature (Tg), crystallinity, Young’s modulus, and density of ester groups. All studied polyesters are commonly used in tissue engineering. PCL and PLLA are FDA-approved polymers. Among aliphatic polyesters used, PLCL has been reported to be the most promising candidate for tissue engineering, surpassing both PLLA and PCL, in some of their properties. The most crucial point is that by the association of PLLA with PCL into PLCL, it is possible to avoid the brittle behavior of PLLA and the low stiffness of PCL [31,32], as well as to reduce the local acidification due to the degradation of PLLA [33]. In general, it may be concluded that PLCL is the most suitable polymer for various tissue engineering applications, particularly when adjustable elasticity and degradability are required. PLLA and PCL differ additionally in the kinetics of degradation, which is relatively fast for PLLA and slow for PCL. PLCL offers an adjustable degradation rate adapted to its use in tissue engineering, tailorable by changing the PCL/PLLA ratio [34].

The main aim of the research was a comparison of polyesters in terms of concentration of gelatin and nitrogen on the surface, the stability of gelatin coating, the impact of the modification on wettability and mechanical properties, and L929 cell–scaffold interaction. This type of modification was compared with the physisorption of gelatin obtained by simple immersion of nonwoven in the protein solution.

**Scheme 1:**
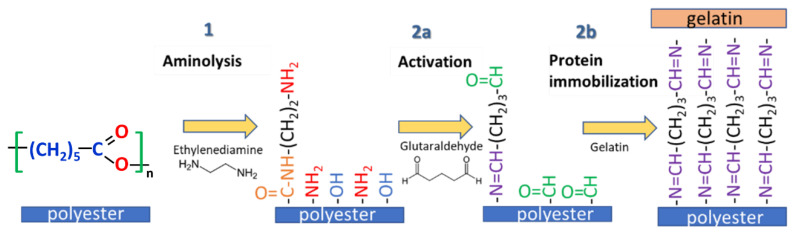
Aminolysis-based immobilization of gelatin.

Scheme 1 illustrates the mechanism of gelatin immobilization based on aminolysis and glutaraldehyde cross-linking that was utilized in this study.

## 2. Materials and Methods

### 2.1. Materials

Three polyesters were used: PCL (Sigma-Aldrich, St. Louis, MO, USA, $\bar{M_{w}}$= 108 kDa, $\bar{M_{n}}$ = 78 kDa, T_g_ = −60 °C), PLCL 70:30 (Resomer^®^ LC703S, Evonik, Essen, Germany, inherent viscosity = 1.3–1.8 dL/g, $\bar{M_{w}}$= 88 kDa, $\bar{M_{n}}$ = 59 kDa, T_g_ = 32–42 °C) and PLLA (PL49, Corbion, Amsterdam, the Netherlands, inherent viscosity = 4.9 dL/g, $\bar{M_{w}}$= 338 kDa, $\bar{M_{n}\;}$ = 197 kDa, T_g_ = 58 °C). Weight and number average molecular weights were determined by us earlier [35]. Solvents for the electrospinning process—acetic acid (AA) (purity degree 99.5%), formic acid (FA) (purity degree ≥ 98%), and hexafluoroisopropanol (HFIP) (purity degree 98.5%)—were purchased from Poch (Gliwice, Poland), Sigma-Aldrich (St. Louis, MO, USA), and Iris Biotech GmbH (Marktredwitz, Germany), respectively. Ethylenediamine (purity degree 99.5%), isopropanol (purity degree 99.8%), ninhydrin (purity degree 99%), ethanol (purity degree 99.8%), and glutaraldehyde (conc. 25%) were purchased from Chempur (Piekary Śląskie, Poland). Gelatin (G1890, type A, gel strength ~300 g Bloom), BCA assay kit (QuantiPro™, for 0.5–30 µg/mL protein conc.), and formamide (≥99.5% (GC), BioReagent, for molecular biology) were procured from Sigma-Aldrich (USA). Diiodomethane (99%+, stabilized) was purchased from Acros Organics (Geel, Belgium).

### 2.2. Preparation of Electrospun Fibers

Three types of nonwovens were obtained using electrospinning equipment (Bioinicia, Spain) in horizontal mode. Polymer solutions (15% *w*/*w* solution of PCL in a mixed solution of acetic acid and formic acid at 9:1 ratio, 7% *w*/*w* PLCL solution in HFIP, and 3.5% *w*/*w* solution of PLLA in HFIP) were pumped through two stainless steel needles at a speed of 6 mL/h (3 mL/h per needle). Other process parameters were as follows: distance between needle and collector 15 cm, collector rotating speed 300 rpm, voltage applied to collector −2 kV, voltage applied to needles PCL 13–15 kV, PLCL 12–14 kV, PLLA 12–14 kV, temperature and humidity conditions PCL 24 °C, 40%, PLCL 38 °C, 40%, PLLA 24 °C, 40%, volumes of polymer solution used to obtain 12 × 31 cm^2^ nonwoven PCL 13 mL, PLCL 18 mL, and PLLA 39 mL.

### 2.3. Chemical Immobilization of Gelatin

Two sets of aminolysis conditions, mild and strong, were applied for each type of fibers to provide different concentrations of free NH_2_ groups and investigate their correlation with the concentration of immobilized gelatin. Conditions and sample designations were chosen based on our previous study on the aminolysis of electrospun fibers [35] and are shown in Table 1.

Samples of 5.5 × 5.5 cm^2^ size were immersed in 80 mL of a given solution of ethylenediamine (EDA) in isopropanol. The process was carried out in an orbital shaker-incubator. After aminolysis, samples were washed three times in deionized water. Aminolyzed samples were subjected to glutaraldehyde treatment in 1% *w*/*v* solution of the reagent in water (80 mL per sample) by 2.5 h at 25 °C. After that, they were washed three times in deionized water. Then, samples were immersed in a 0.2% *w*/*v* solution of gelatin in water (80 mL per sample). The process was conducted in an orbital shaker-incubator at 38 °C by 20 h. After this treatment, the samples were washed in deionized water at 38 °C for 24 h to remove weakly bound gelatin, and dried under a fume hood.

Unmodified samples were used as controls and are marked as Name-of-polymer_Control.

Additionally, to examine gelatin concentration as a function of the concentration of free NH_2_ groups, PLCL fibers were subjected to a wide range of aminolysis conditions. Three concentrations of EDA, 2, 6, and 10% (*w*/*v*), and four time points, 5, 10, 15, and 30 min, were applied. The temperature was set at 30 °C. The process of gelatin immobilization was the same as described above.

### 2.4. Physisorption of Gelatin

In parallel to chemical immobilization, polymer samples were subjected to the physisorption of gelatin. Firstly, they were immersed in an isopropanol/water (50:50, *v*/*v*) solution for 0.5 h. Then, they were washed three times in water. After that, samples were immersed in a gelatin solution. Process parameters for gelatin immobilization were the same as in the case of chemically modified samples, including washing and drying procedures. Sample designations are presented in Table 1.

### 2.5. Sample Imaging

Scanning electron microscopy (SEM, Jeol JSM-6010PLUS/LV InTouchScope™) was used for imaging the samples after modification. Before imaging, samples were coated with gold. The acceleration voltage was in the range of 7–10 kV.

### 2.6. Gelatin Concentration on Fibers’ Surface

The concentration of gelatin on fibers’ surface was determined using BCA assay with BCA solution prepared according to producer recommendation. Three samples (1–1.3 mg) of each modified material were put into Eppendorf vials. 0.5 mL deionized water and 0.5 mL BCA solution were added. Samples were incubated for 2 h at 37 °C. To obtain a calibration curve, 0.5 mL gelatin solution (concentrations 5–30 µg/mL) was mixed with 0.5 mL BCA solution and incubated at the same conditions. The absorbance was read at 562 nm using a spectrophotometer (Thermo Fisher Scientific MultiskanGo, Waltham, MA, USA). Results were presented as the concentration of gelatin on the surface in µg/mg of fibrous sample.

### 2.7. Analysis of Chemical Structure—X-ray Photoelectron Spectroscopy

Five samples were chosen to be analyzed by X-ray photoelectron spectroscopy: PCL_Control, PCL_Chem_I, PCL_Chem_II, PCL after stronger aminolysis corresponding to PCL_Chem_II reaction parameters (marked as PCL_Am_II), and a fibrous gelatin sample. The gelatin sample was prepared by electrospinning (parameters: 5% solution in HFIP, voltage applied to needle 10 kV, grounded collector, distance between needle and collector 15 cm, and solution flow rate 0.5 mL/h).

For XPS measurements using non-monochromatic Mg K_α_ radiation (1253.6 eV), the samples were inserted into the spectrometer (SPECS GmbH, Berlin, Germany) without further treatment. The samples were studied under a vacuum of 5 × 10^−8^ mbar at room temperature. After measuring overview spectra of the samples at a constant analyzer pass energy (E_pass_) of 50 eV, individual 1s spectra of C, N, and O were recorded with higher resolution with E_pass_ = 13 eV. This corresponds to a spectrometer resolution of 1.0 eV (FWHM of the Ag 3d_5/2_ line). The electrostatic sample charging was corrected by setting the binding energy of C 1s electrons of aliphatic carbon to 285.0 eV. The XPS spectra were analyzed using the CasaXPS software package. The error of the binding energies is ±0.1 eV. The error of relative intensities is about ±5%.

### 2.8. pH Measurements

The pH was measured using a pH meter (CP-401, Elmetron, Zabrze, Poland) and electrode ERH-NS type (Elmetron, Poland). A 10 mL volume of 0.2% gelatin solution in distilled water was prepared in triplicate for measurement of pH change of blank solution and solutions with immersed PCL, PLCL, and PLLA samples. The sample size corresponded to the sample/solution ratio used in the immobilization process. For each solution, pH was measured before immersion of the sample and after 20 h of sample incubation at 38 °C.

### 2.9. Wettability

Wettability was determined using a goniometer (Data Physics OCA 15EC, Filderstadt, Germany). Waterdrop dosing volume and dosing rate were equal to 1 µL/s and 2 µL/s, respectively. Due to the complete wettability of samples, the time of waterdrop absorption was measured. Measurements were repeated 5 times for each sample.

### 2.10. Surface Free Energy

Total surface free energy and its components were calculated for polymer films. Films were obtained by solvent casting technique from the same solutions as in the case of the electrospinning process. Contact angles were measured using three liquids: distilled water, formamide, and diiodomethane. Measurements were repeated 5 times. Surface free energy was calculated using SCA20 (v.5.0.10, DataPhysics Instruments GmbH, Filderstadt, Germany) software with the Owens, Wendt, Rabel and Kaelble (OWRK) model.

### 2.11. Mechanical Testing

A uniaxial testing machine, Lloyd EZ-50 (USA), was used to determine Young’s modulus, stress at break, and strain at break. Three samples (5 × 40 mm^2^, testing area 5 × 20 mm^2^) for each type of material were tested. The machine was equipped with handles for thin and delicate samples with a 50 N load cell. Cross-head speed was set at 5 mm/min. The thickness of each sample was measured with a thickness gauge.

### 2.12. Stability of Gelatin Coating

Stability of the gelatin layer on the fiber surface was tested up to 90 days by incubation in phosphate-buffered saline (PBS). Taking into consideration that polymer degradation can strongly affect measurements, PCL fibers, having the longest degradation time, were chosen for the test. It was presented previously that PCL fibers do not lose their mass during 90-day immersion in PBS solution [36]. The samples were additionally weighted showing no weight loss during the incubation test. Three samples of PCL per time point were tested. Samples were immersed in 10 mL of the PBS solution with the addition of sodium azide (0.1% *w*/*v*) inhibiting bacteria growth. Samples were incubated at 37 °C and shaken once every few days. The stability of the gelatin layer was evaluated on the basis of gelatin concentration measurements using a BCA assay. The BCA test was conducted according to the procedure described above.

### 2.13. Cell–Scaffold Interaction

Mouse fibroblast cells (L929, Sigma Aldrich) were used to evaluate cell response. Dulbecco’s modified Eagle’s medium (DMEM, Thermo Fisher Scientific) was supplemented with fetal bovine serum (FBS, Thermo Fisher Scientific) to a concentration of 10% *v*/*v* and penicillin–streptomycin (Thermo Fisher Scientific) to a concentration of 1% *v*/*v*.

Samples were sterilized by immersion in 70% solution of ethanol in water and 30 min exposure to UV light on each side. Then, they were placed in a 48-well culture plate—three samples for the cell metabolic activity test and two samples for SEM observation for each type of material. A total of 2 × 10^3^ cells were seeded per well. Cell culture was performed in an incubator at 37 °C in 5% CO_2_ for 3 and 5 days.

PrestoBlue™ (Thermo Fisher Scientific) test was used to investigate cell metabolic activity. The culture medium was removed, and each sample was rinsed with PBS (Thermo Fisher Scientific). Then, 180 µL of PBS and 20 µL of PrestoBlue reagent in the PBS solution were added to each well. PrestoBlue reagent was also added to pure PBS as a control. The plate with samples was put into an incubator for 40 min to enable reaction. After this time, 100 µL of each solution was transferred to a 96-well plate. The emission of light was measured using Fluoroskan Ascent (Thermo Scientific) at an excitation light wavelength of 530 nm and emission light wavelength of 620 nm. Results were calculated to percentage metabolic activity using tissue culture polystyrene as a 100% control.

Samples used for SEM observation were gently rinsed with PBS twice and then fixed with 2.5% glutaraldehyde/PBS overnight. Dehydration was performed using ethanol in water solution series (30, 40, 50, 60, 70, 80, 90, 100 (×2)% *v*/*v*), hexamethyldisilazane (HDMS, Sigma Aldrich)/ethanol solutions (1:2 and 2:1 *v*/*v*), and pure HDMS. Each ethanol solution was removed after 20 minutes, and HDMS was left overnight. All samples were imaged in comparison to TCP (Tissue Culture Plastic) using scanning electron microscopy (SEM, Jeol JSM-6010PLUS/LV InTouchScope™). Before imaging, samples were coated with gold. The acceleration voltage was in the range of 8–10 kV.

Fluorescence microscopy (FM) imaging was performed after immunohistochemical staining. Cells were rinsed with PBS, fixed in 4% paraformaldehyde, kept in 0.1% Triton-X for 5 min, and rinsed again with PBS. The actin skeleton and nucleus were stained with ActinGreen™ and NucBlue™ Reagent (Thermo Scientific), respectively. All samples and TCP were imaged using a fluorescent microscope (Leica DMI3000B, Leica Microsystems, Wetzlar, Germany).

### 2.14. Statistical Analysis

Dependent sample *t*-test and Wilcoxon test were performed on pH data. One-way analysis of variance (ANOVA) followed by NIR post hoc test was performed on stress and strain at break, and metabolic activity data using Statistica (v.13, Tibco Software Inc., Palo Alto, CA, USA) software. Significance was set at *p* < 0.05.

## 3. Results and Discussion

### 3.1. Fiber Morphology

**Figure 1:**
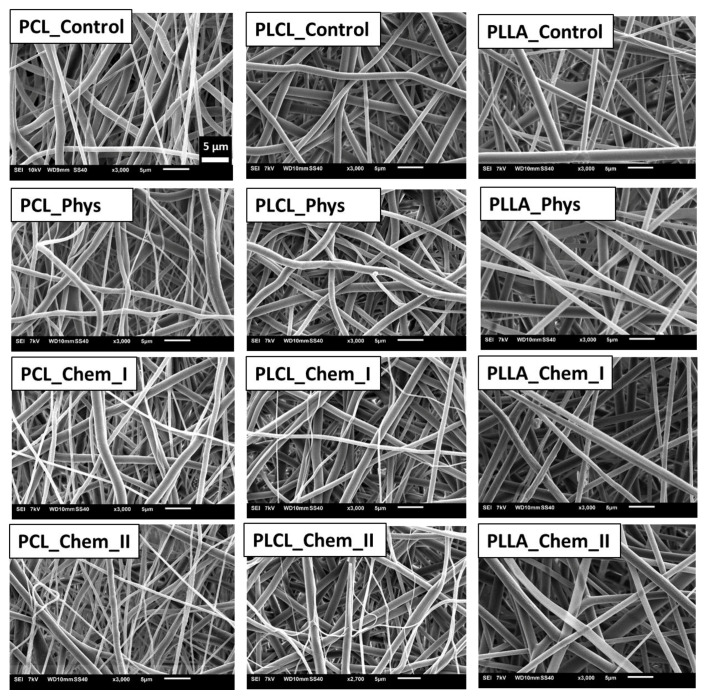
Morphology of controls and samples subjected to physisorption and chemical immobilization of gelatin.

**Figure 2:**
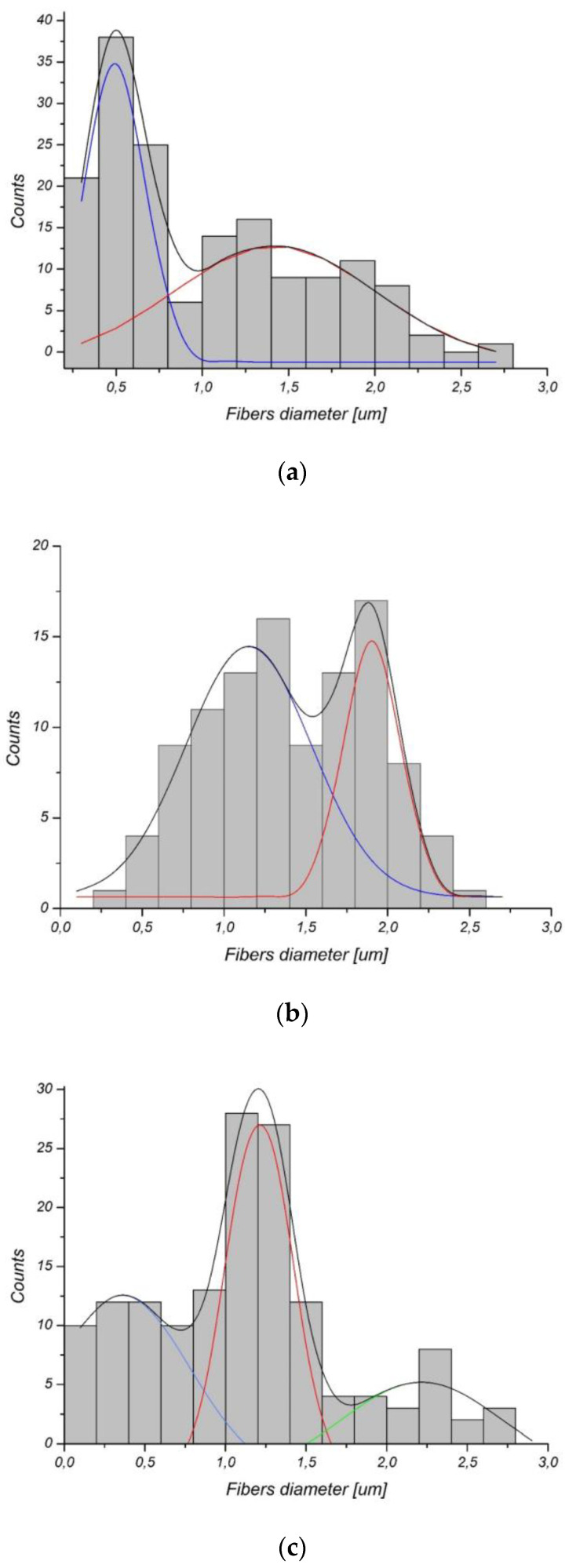
Fiber diameter histograms of (**a**) PCL, (**b**) PLCL, (**c**) PLLA.

As shown in Figure 1, the process of gelatin immobilization either by physical adsorption or covalent conjugation did not result in a change in fiber morphology. It is well known that aminolysis can cause fiber cracking and fragmentation; however, on the basis of our previous study on aminolysis, conditions that maintain fiber morphology were carefully chosen [35]. It was also observed that glutaraldehyde activation and gelatin immobilization did not affect fiber morphology. The only exception is a PLCL_Chem_II sample, for which morphology change—single fiber breaking in some regions of the sample was recognized. It is the effect of the aminolysis reaction and mechanical stress during further treatment. In Figure 2, histograms of fiber diameter for control samples are shown. PCL and PLCL have a bimodal distribution of fiber diameter, and in the case of PLLA, the distribution is trimodal. It is evident that the low diameter fraction of the histogram dominates over the high diameter fraction for PCL with its most probable value around 600 nm. This is the opposite of the situation observed for PLLA with a large fraction of high fiber diameter. This difference in distributions implies a difference in specific surface area, which can affect the amount of immobilized gelatin.

### 3.2. Detection of Gelatin on Fiber Surface

**Figure 3:**
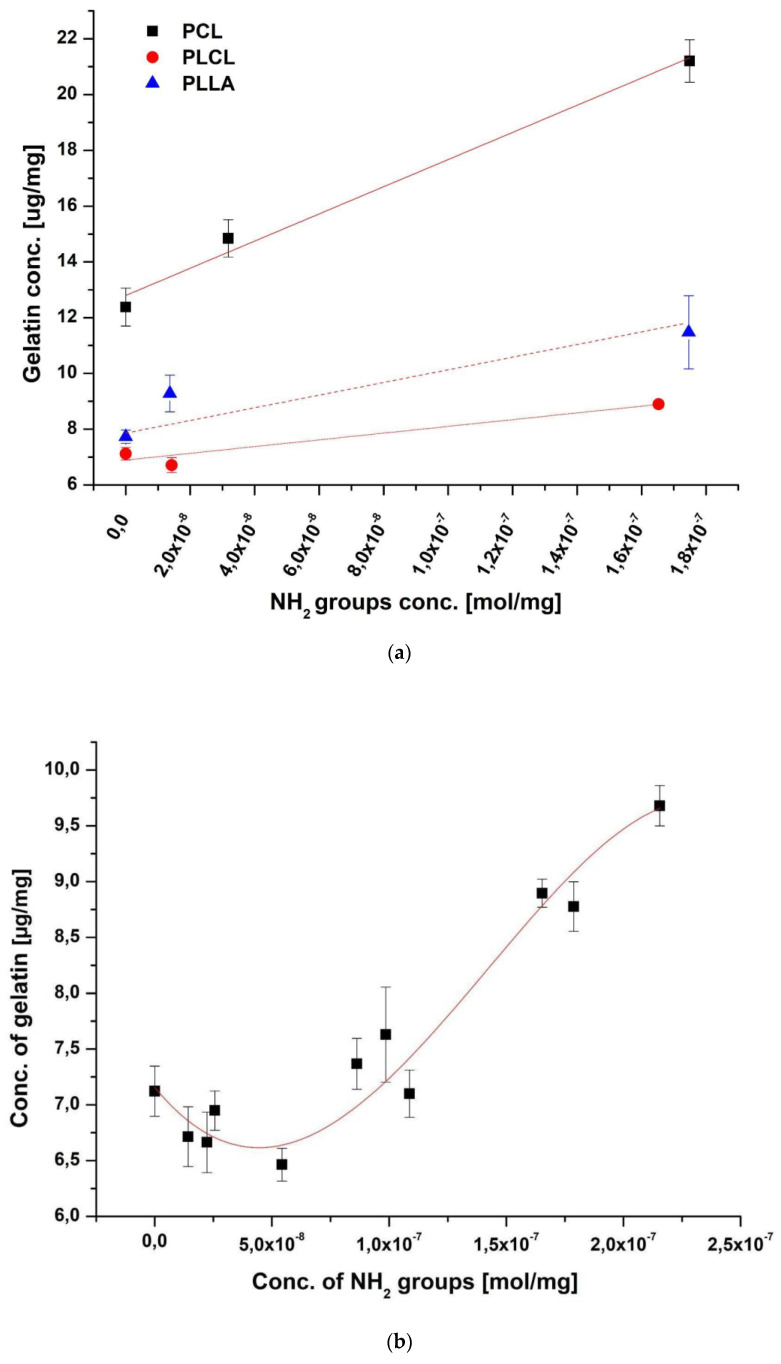
Concentrations of gelatin on the surface of the fibers as a function of free NH_2_ groups concentration for (**a**) all investigated polymers (the subsequent amine concentration points on X-axis correspond to physical adsorption, mild chemical and strong chemical immobilization) and (**b**) PLCL fibers.

Figure 3a presents the concentration of gelatin as a function of free NH_2_ groups. The first observation is that despite similar concentrations of NH_2_ groups, gelatin concentrations vary widely between polymers, being the highest for PCL and the lowest for PLCL. Moreover, it is seen that the difference in gelatin concentration between polymers starts at zero of amine groups (physical adsorption). This fact together with the highest slope of the PCL curve indicates the easiest immobilization of gelatin, both physical and chemical on this polymer. This relatively high physical adsorption is consistent with previous observations by B. Atthoff, J. Hilborn who showed that spontaneous hydrolysis of degradable aliphatic polyesters surface in aqueous media is sufficient for protein adsorption [37]. The unexpectedly high physisorption of gelatin is most probably a result of various molecular interactions, including ionic and hydrophobic interactions as well as interactions with carboxyl and hydroxyl groups created on the polyester surface by spontaneous hydrolysis in an aqueous medium during gelatin immobilization. The fundamental explanation of the interactions involved in protein adsorption is included in the review of J. D. Andrade and V. Hlady [38]. Considering the simplicity of physical adsorption, this type of modification could be very attractive. On the other hand, physical adsorption could be not stable or reversible, which is confirmed by our results, presented in the following part of our study.

The pH of gelatin solution during immobilization was measured to exclude the impact of pH change due to possible polymer degradation on subsequent difference in gelatin adsorption. As shown on the Figure S1 there is no significant difference in pH before and after immobilization.

Surface energy was measured additionally for polymer films as shown in Table 2. In fact, the polar component is the lowest for PCL film, and it is observed by other authors that protein adsorption is higher on more hydrophobic surfaces [39,40]. However, it has to be taken into consideration that factors such as micro/nanoroughness, and differences in fiber diameter distribution can also influence the concentration of immobilized gelatin.

Irrespective of the highest content of immobilized gelatin on PCL due to the highest sensitivity to both physical and chemical sorption, one should be aware that the first step of modification, aminolysis, needs much more time in the case of PCL compared to PLCL and PLLA (Table 1). It can be explained by a combination of several factors—the differences in ester bond density, glass transition temperature, and crystallinity (surface crystallinity) among investigated polymers [41].

A more detailed analysis of the effect of amine groups concentration on gelatin immobilization was performed for PLCL (Figure 3b). Results support the previous observation that there is an increase of immobilized gelatin concentration with the concentration of amine groups. However, contrary to the main rising trend, there is a systematic, rather small reduction of immobilized gelatin concentration with amine concentration below 5 × 10^−8^ mol/mg of amine groups. It should be noted that most of the immobilized gelatin in the range of such small amine concentrations is due to physical adsorption, so the observed local reduction of immobilized gelatin with increasing amine concentration in this concentration range indicates some mechanisms from the side of aminolysis, which leads to reduction of the physically adsorbed layer. This observation is unexpected and needs further investigation.

**Figure 4:**
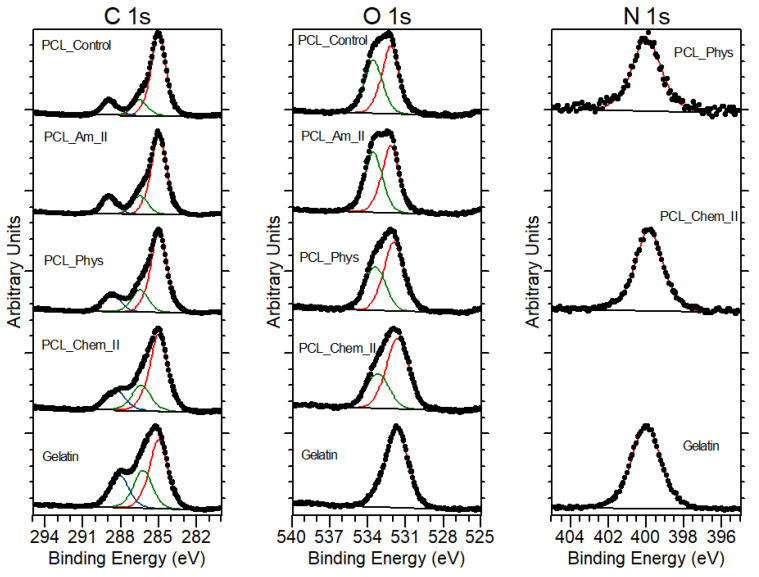
XPS spectra of PCL_Control, PCL_Am_II, PCL_Phys, PCL_Chem_II and gelatin.

Table 3 shows atomic percentages for PCL samples before modification, after aminolysis, after physical and chemical modification with gelatin, and for gelatin calculated from XPS analysis. Oxygen and carbon concentrations (atom %) for the PCL control sample agree within the experimental uncertainties with the expected stoichiometric values, which are 25% and 75%, respectively. For aminolyzed samples, there is no evidence of N 1s peak from nitrogen, indicating that its concentration is below the detection limit of XPS. However, the introduction of NH_2_ groups was confirmed by the ninhydrin test. The presence of nitrogen was detected in the case of PCL samples with physisorbed and chemically bound gelatin. Atomic concentrations of nitrogen are 5% for PCL_Phys and 8% for PCL_Chem_II. This correlation clearly corresponds to the results obtained from the BCA test. Nitrogen concentration for the control sample of electrospun gelatin is equal to 16%, which indicates that the gelatin layer is thinner than 10 nm (depth of XPS analysis) for both PCL samples. The decomposition of C 1s and O 1s peaks is shown in Table 4. The calculated percentage of bonds for the PCL_Control sample corresponds to the data obtained by other authors [42,43]. Aminolysis did not change the initial contribution of bonds. In the case of samples coated with gelatin, it is evident that the contribution of bonds changes towards values adequate for the gelatin control sample. XPS spectra of studied samples are shown on Figure 4.

### 3.3. Wettability

**Figure 5:**
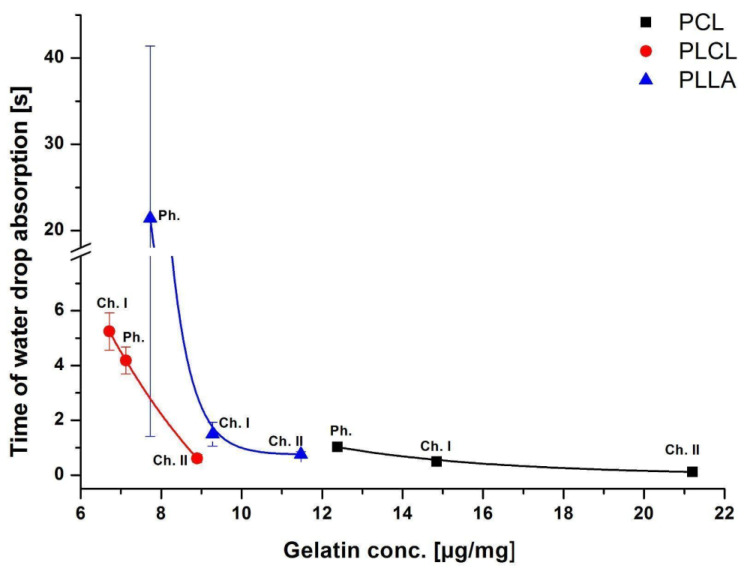
Time of waterdrop absorption as a function of gelatin concentration for physically (Ph.) and chemically (Ch. I; Ch. II) modified samples.

Our analysis of wettability clearly indicates that all samples with immobilized gelatin were completely wettable, contrary to the hydrophobic character of control polymer scaffolds. Water contact angles were equal to 135.69 ± 3.02°, 131.05 ± 1.66°, and 132.90 ± 2.48° for PCL, PLCL, and PLLA, respectively, as shown in our previous study [35]. In order to compare the wettability of gelatin-coated samples, the time of waterdrop absorption was measured (Figure 5). Increase of sample wettability after surface modification origins from exposed hydrophilic groups of immobilized protein. For polymer films coated with proteins, rather a decrease of water contact angle than a complete wettability is observed [46,47,48]. Complete wettability after protein immobilization is reported for nonwovens and appears to be additionally associated with the capillary effect of fibrous architecture [49]. It is worth noting that water absorption ability is important for nutrition transport through the scaffold [50]. It can also accelerate the hydrolytic degradation of the scaffold [51].

From our results, it is clear that for all polymers the time of absorption is correlated with the concentration of gelatin on fibers’ surface being the lower the higher the gelatin concentration. In the case of PCL samples, with the highest concentrations of gelatin among all studied fibers, there was only a slight difference in time of absorption. A wide range of the absorption time values for PLLA_Phys suggests that the physisorbed layer of gelatin could be inhomogeneously distributed on fibers, in contrast to the chemically bound coating. However, there was no such observation for PLCL or PCL fibers. Inhomogeneity of physisorbed coatings was reported previously by others [42].

### 3.4. Mechanical Properties

**Figure 6:**
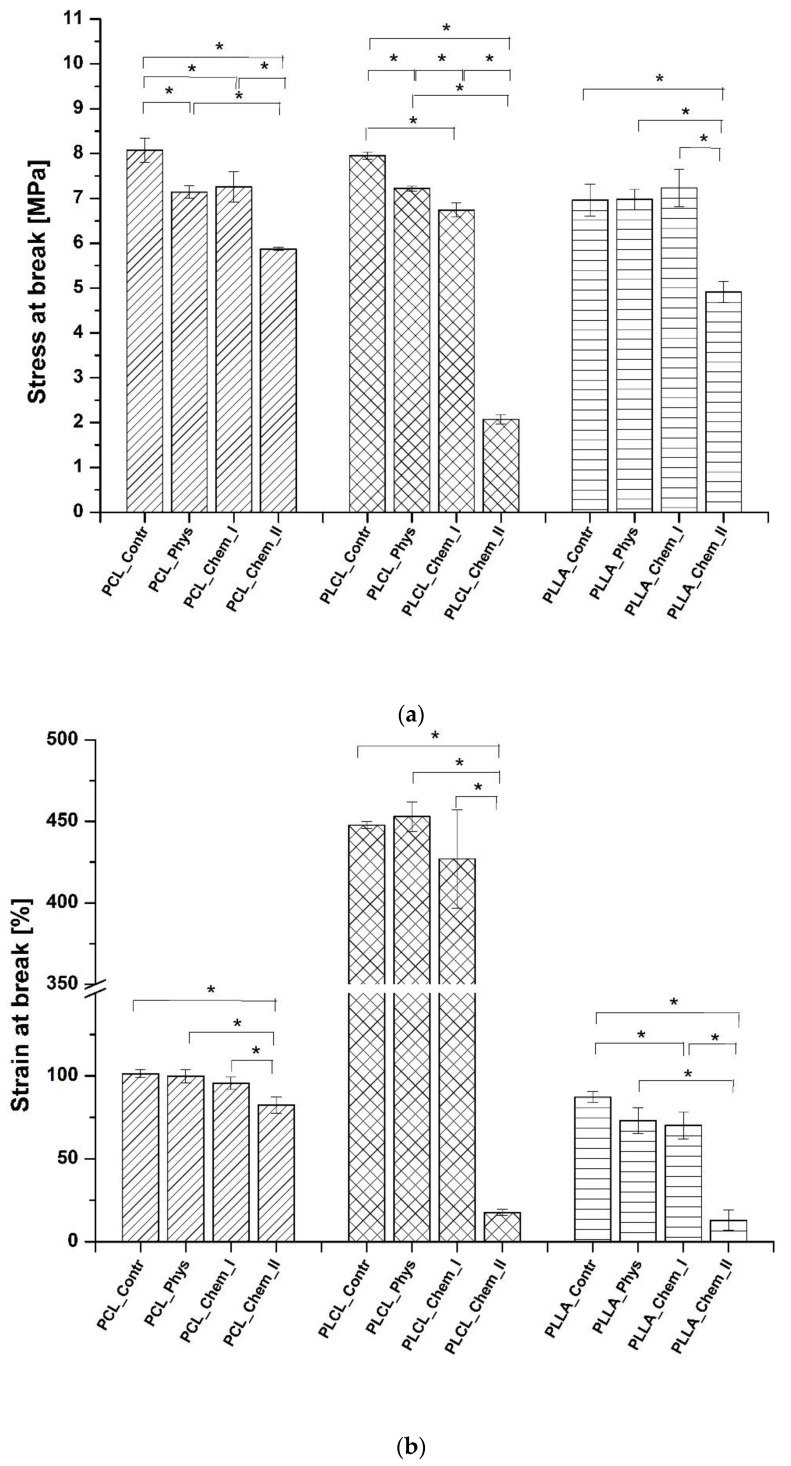
Mechanical properties: (**a**) stress at break, and (**b**) strain at break of control and gelatin-coated fibers. Statistically significant differences were marked as * (*p* < 0.05).

Stress at break and strain at break of all samples before and after modifications were shown in Figure 6, and representative stress–strain profiles are presented in Figure S2. Only a slight decrease or no change in stress at break was observed for physisorbed and mildly chemically modified samples. A slight decrease for physically modified samples is most probably related to a slightly elevated temperature during gelatin immobilization (38 °C). For all polymers, a more significant decrease was identified in the case of a stronger chemical modification, especially for PLCL_Chem_II. Stress at break is clearly affected by aminolysis reaction, as discussed in our previous work [41].

Similar correlation was observed in the case of a strain at break. Only a slight decrease or no change in the strain at break was observed in the case of all samples with physisorbed gelatin or those that were mildly modified via aminolysis. The high decrease was identified for more aggressively treated samples, especially for PLCL_Chem_II and PLLA_Chem_II. It is worth noting that all PCL samples indicated a relatively low decrease in stress and strain at break.

It should be also emphasized that for all studied nonwovens, conditions that were applied in the case of mild aminolysis, enable for maintaining mechanical properties on a similar level as in the case of physisorption. Considering results for samples after stronger chemical modification, it has to be taken into account that aminolysis is also applied to obtain fragmented fibers that can be then modified with proteins and immersed in hydrogels to mimic the extracellular matrix [52,53]. In this application, their mechanical properties are not as important as in the case of fibrous scaffolds. In the case of Young’s modulus, there was no common correlation between values of modulus and type of modification. In the literature, there are contradictory observations of aminolysis impact on Young’s modulus [54,55].

### 3.5. Coating Stability

**Figure 7:**
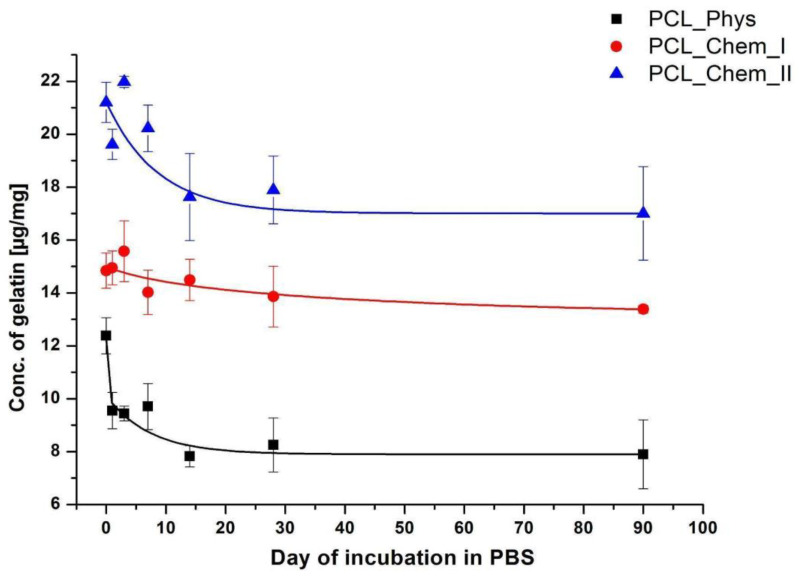
Concentration of gelatin on the PCL fibers as a function of incubation time in PBS for various time points (0; 1; 3; 7; 14; 28; 90 days).

An important feature that can determine the applicability of a given modification is the stability of the coating. The stability of the gelatin layer on the PCL samples after incubation in PBS solution is shown in Figure 7. For physically and strongly chemically modified samples, the most significant loss was observed up to 14 days of incubation. Then, the concentration of gelatin is stable, even after 90 days. The highest loss is observed for the sample with physisorbed gelatin approaching almost 37% after 14 days of incubation. This loss is much lower for chemically modified samples, particularly for PCL_Chem_I, for which 2.4% loss was noticed on the 14th day. In the case of PCL_Chem II, the loss of gelatin was equal to 16.9% at this time point. It is highly probable that major loss of gelatin from the surface of chemically modified fibers originates from physisorbed gelatin. It is worth noting that there was no weight loss of PCL samples during the incubation test, which could disturb the measurements.

Interestingly, even after 90 days of incubation, the gelatin layer was still present on the surface of the physically modified sample (more than 60% of initial gelatin concentration), which shows how strong are physical interactions between gelatin and polymer. In the literature, lower stability of coating is frequently pointed out as a drawback of using physisorption for protein immobilization [7]. Our results indicate saturation of desorption process after certain time. However, it may be different in in vivo conditions. Protein adsorption and desorption are complex processes and the mechanisms are still not completely understood [56,57]. Thus, in our opinion, coating stability should be always considered from the perspective of given biomolecule, substrate, and the application site.

### 3.6. Biological Evaluation

**Figure 8:**
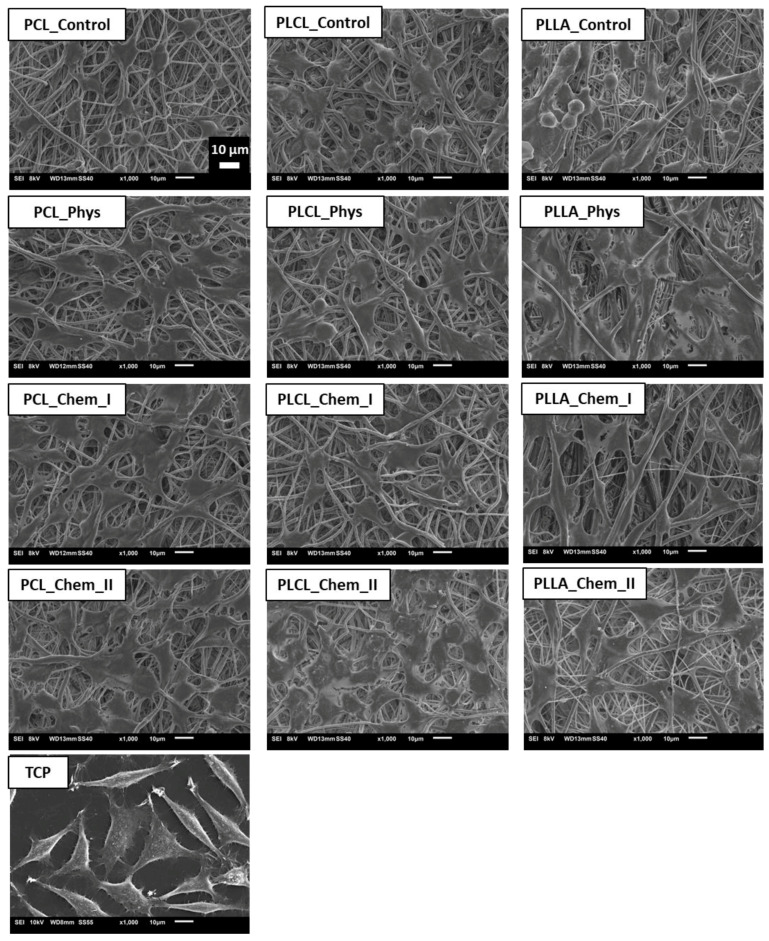
L929 cells cultured for 5 days on control and gelatin-coated samples observed with scanning electron microscopy.

**Figure 9:**
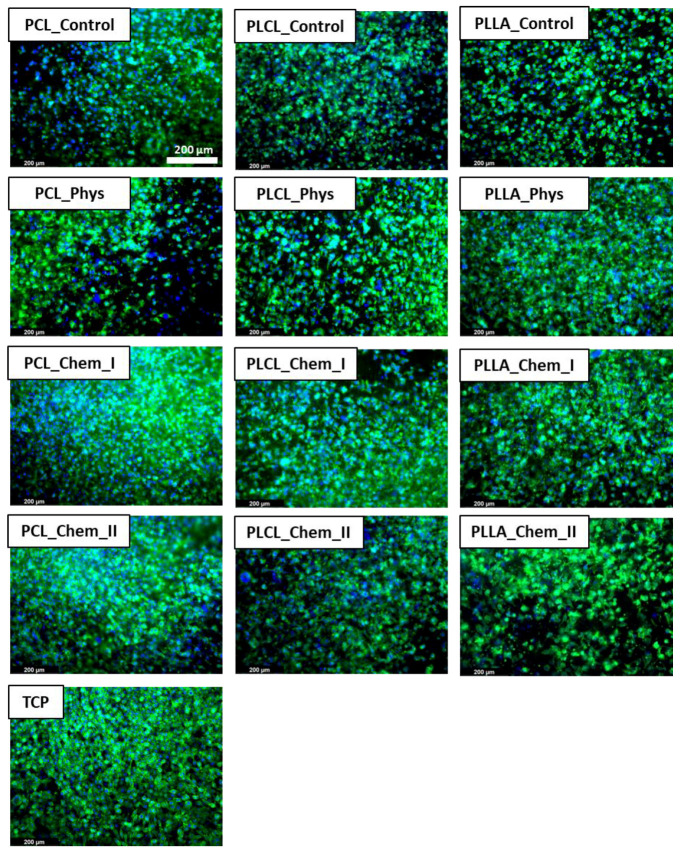
L929 cells cultured on control and gelatin-coated samples for 5 days observed with fluorescence microscopy.

**Figure 10:**
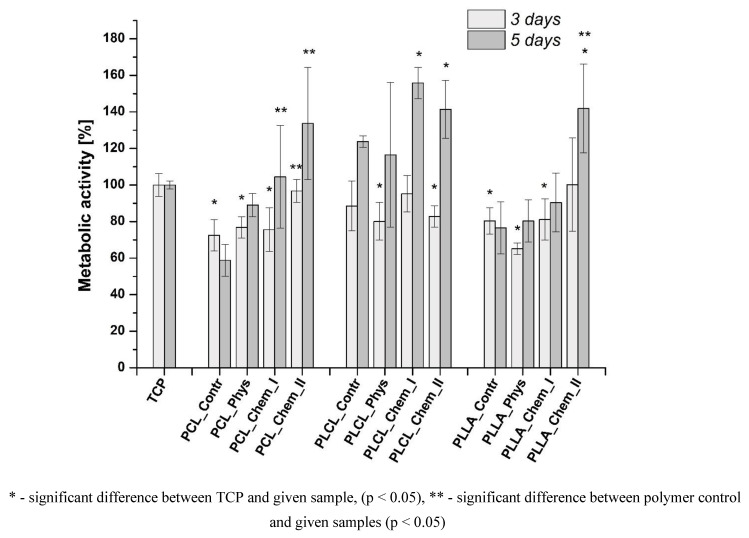
Viability of L929 cells cultured on control and gelatin-coated samples.

The morphology of L929 cells imaged with scanning electron microscopy is presented in Figure 8. For all samples, improvement of morphology after gelatin immobilization was noticed. On the control samples, rounded and not spread cells are present. In the case of both physically and chemically coated samples, expansion of cells is observed, which indicates better interaction of L929 cells with materials. In the case of PCL and PLCL, the best morphology, characterized by stretched filopodia, was observed after stronger chemical modification. For PLLA, there is no significant difference between modified samples. Fluorescence microscopy confirmed the previous observation (Figure 9). Actin spreading proves improvement of cell–material interaction after gelatin immobilization. There is also improvement in cell metabolic activity after gelatin immobilization, especially after 5 days of culture (Figure 10). Gradual increase is particularly observed for PCL samples. In addition, for PLLA_Chem_II enhancement of metabolic activity was observed. This increase can result from simply a higher concentration of gelatin, and/or better gelatin conformation in the case of chemically modified samples [58]. It is also worth noting that initial metabolic activity for PLCL was higher than for PCL and PLLA controls, thus the effect of gelatin immobilization could be not so significant for PLCL.

## 4. Conclusions

In this study, two types of modification—physisorption and chemical immobilization—of gelatin were compared for PCL, PLCL, and PLLA electrospun nonwovens. It was shown on the basis of the BCA test and the XPS analysis that chemical immobilization could provide a higher concentration of gelatin on the fiber surface. However, physisorption provides a relatively high concentration of gelatin, especially in comparison to samples subjected to mild chemical immobilization. On the basis of XPS results, the thickness of both physically and chemically bound gelatin layers was estimated to be less than 10 nm. Initially, hydrophobic samples were completely wettable after each type of modification and the time of waterdrop absorption was correlated with the gelatin concentration. Mild chemical immobilization did not change significantly mechanical properties of samples, likewise physisorption. Aminolysis conditions that were applied to provide a higher concentration of free amine groups result in a significant decrease of stress and strain at break in the case of PLCL and PLLA samples. Thus, modification method should be chosen depending on the application site and taking into account its impact on mechanical performance. It is worth emphasizing that PCL samples were much more resistant to loss of mechanical toughness than PLCL and PLLA samples. Incubation tests showed that chemically immobilized gelatin layer is more stable than physisorbed; however, for physically modified surfaces only partial desorption was observed, even after 90 days of immersion in PBS. L929 cell imaging and the results of metabolic activity test indicate a positive effect of both physical and chemical modification on cell–scaffold interaction with the better cell response on chemically modified samples, which could result from simply a higher concentration of gelatin, or better gelatin conformation. However, there is a difference in the intensity of the effect of gelatin coating on cell response between all studied polymers. The clearest trend of improvement was observed in the case of PCL fibers. Thus, in our opinion, the immobilization method should be always considered from the perspective of given biomolecule, substrate, and type of application.

## Figures and Tables

**Table 1:** Studied samples and their aminolysis parameters.

Sample Name	Polymer	EDA/Isopropanol Conc [% *w/v*]	Time	Temp [°C]	Concentration of NH2 Groups [mol/mg] [35]
PCL_Phys	PCL	-	-	-	0
PCL_Chem_I	PCL	10	24 h	30	(3.18 ± 0.31) × 10^−8^
PCL_Chem_II	PCL	30	24 h	30	(1.69 ± 0.19) × 10^−7^
PLCL_Phys	PLCL	-	-	-	0
PLCL_Chem_I	PLCL	2	5 min	30	(1.42 ± 0.08) × 10^−8^
PLCL_Chem_II	PLCL	10	10 min	30	(1.65 ± 0.15) × 10^−7^
PLLA_Phys	PLLA	-	-	-	0
PLLA_Chem_I	PLLA	2	5 min	30	(1.36 ± 0.13) × 10^−8^
PLLA_Chem_II	PLLA	6	10 min	30	(1.75 ± 0.22) × 10^−7^

**Table 2:** Surface free energies and their components for control samples.

	Dispersive Component [mN/m]	Polar Component [mN/m]	Surface Free Energy [mN/m]
PCL	38.25 ± 4.8	0.98 ± 0.80	39.24 ± 4.13
PLCL	31.96 ± 1.83	6.98 ± 1.23	38.94 ± 0.93
PLLA	35.94 ± 3.75	4.61 ± 0.90	40.54 ± 3.14

**Table 3:** Atomic concentrations (atom-%) of oxygen, carbon and nitrogen for PCL and gelatin samples.

	O	C	N
PCL_Control C_6_H_10_O_2_ PCL (Kołbuk et al.) [44]	22 25 24.9	78 75 75.1 *	0 0 0
PCL_Am_II	23	77	0
PCL_Phys	22	73	5
PCL_Chem_II	22	70	8
Gelatin C_102_H_151_O_39_N_31_ Gelatin (Kołbuk et al.) [44] Gelatin (Jalaja et al.) [45]	19 23 18.9 21.5	65 59 65.2 * 67.0	16 18 15.9 11.5

* calculated as the difference from O% and N%.

**Table 4:** Relative concentrations of functional groups for PCL and gelatin samples.

	C 1s			N 1s	O 1s	
	C-CH_x_R_y_^b^ 285.0 eV ^a^	C-OR ^b^ 286.2–286.5 eV	C=O 288.1–288.9 eV	-NH_2_ 399.8–400.0 eV	O=R 531.6–532.2 eV	O-R 533.2–533.6 eV
PCL_Control	73	14	13	-	56	44
PCL_Am_II	69	16	15	-	53	47
PCL_Phys	67	19	15	100	61	39
PCL_Chem_II	63	21	16	100	67	33
Gelatin	49	28	23	100	100	-

^a^ This C 1s component was used to correct for electrostatic sample charging. ^b^ Hydrocarbon group.

## Data Availability

The raw/processed data required to reproduce these findings cannot be shared at this time due to technical or time limitations.

## Supplementary Materials

The following supporting information can be downloaded at: https://www.mdpi.com/article/10.3390/polym14194154/s1, Figure S1. pH of the gelatin solutions before and after immersion of the polymer samples; Figure S2. Exemplary stress-strain profiles of (a) PCL, (b) PLCL, (c) PLLA.
