# Supplementary material for: Immobilization of Gelatin on Fibers for Tissue Engineering Applications: A Comparative Study of Three Aliphatic Polyesters
Source: Polymers (Basel). 2022 Oct 4;14(19):4154. doi: 10.3390/polym14194154 (PMC9572612; doi:10.3390/polym14194154)
Supplement: Supplementary file 1 [file polymers-14-04154-s001.zip › polymers-1912726-supplementary.pdf]

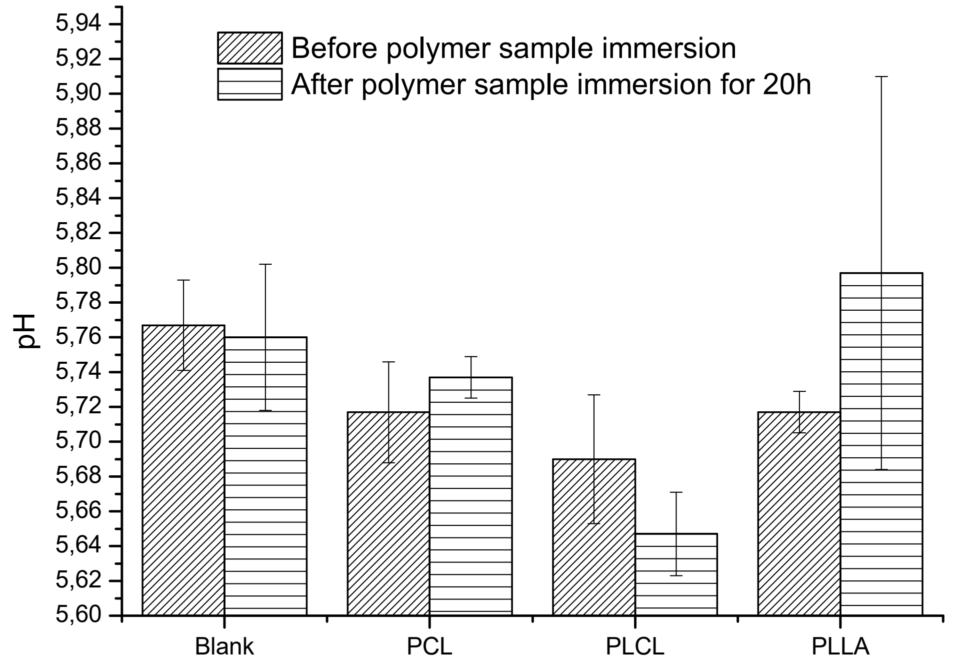

Figure S1. pH of the gelatin solutions before and after immersion of the polymer samples.

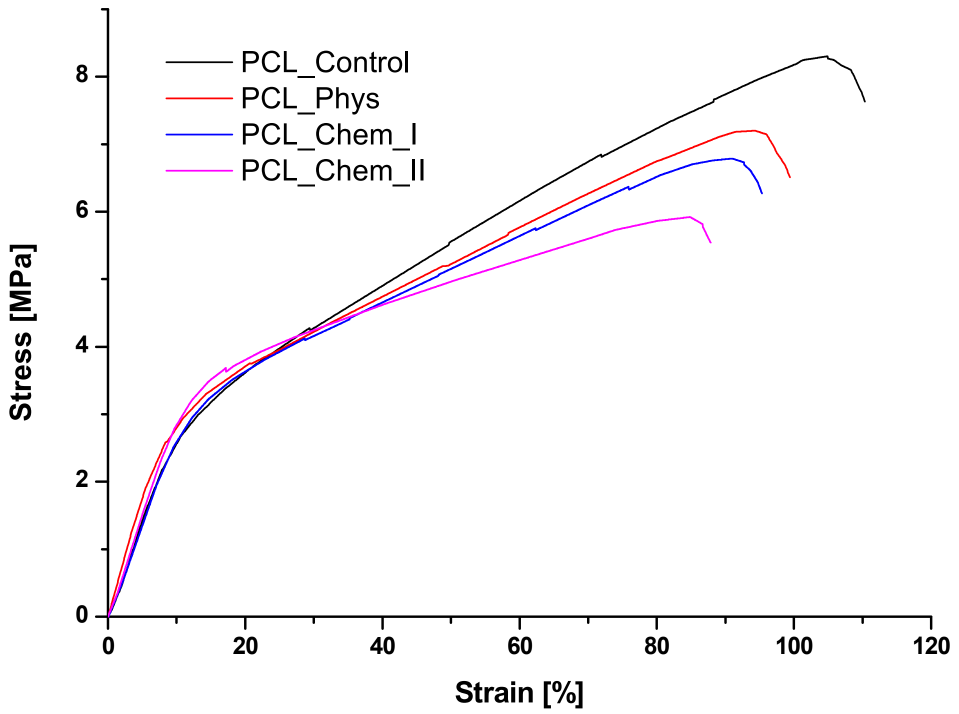

(a)

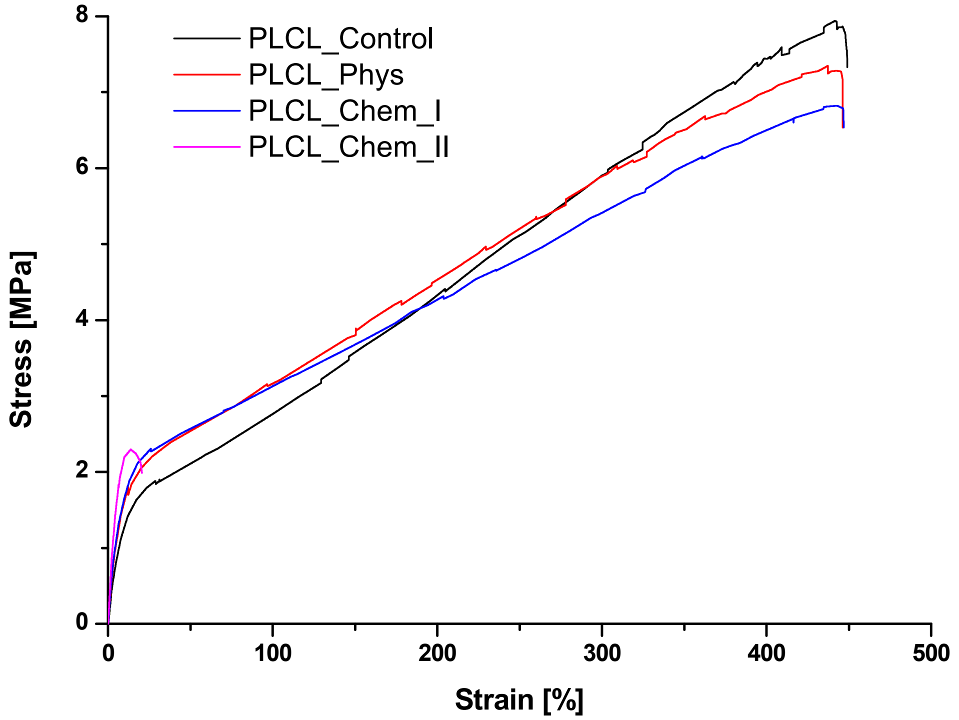

(b)

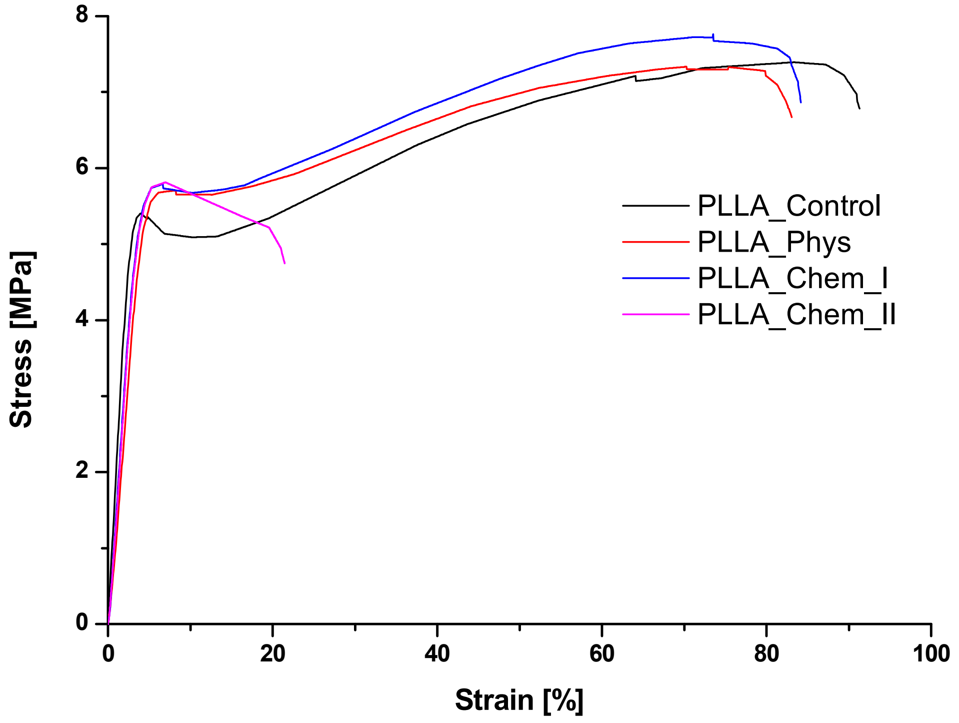

(c)

Figure S2. Exemplary stress–strain profiles of (**a**) PCL, (**b**) PLCL, (**c**) PLLA.
